# Expression and cellular localization of hepcidin mRNA and protein in normal rat brain

**DOI:** 10.1186/s12868-015-0161-7

**Published:** 2015-04-21

**Authors:** Ruma Raha-Chowdhury, Animesh Alexander Raha, Serhiy Forostyak, Jing-Wei Zhao, Simon Russell William Stott, Adrian Bomford

**Affiliations:** John Van Geest Centre for Brain Repair, Department of Clinical Neuroscience, University of Cambridge, Cambridge, UK; Institute of Liver Studies, King’s College Hospital, London, UK; Department of Neuroscience Institute of Experimental Medicine Academy of Sciences of the Czech Republic, Prague, Czech Republic

**Keywords:** Hepcidin, Ferroportin, Defensin, Inflammatory cytokines, Brain iron homeostasis, Blood brain barrier, Pericytes, Sub-ventricular zone, Neurogenesis

## Abstract

**Background:**

Hepcidin is a peptide hormone belonging to the defensin family of cationic antimicrobial molecules that has an essential role in systemic iron homeostasis. The peptide is synthesised by hepatocytes and transported in the circulation to target tissues where it regulates the iron export function of the ferrous iron permease, ferroportin. In the brain hepcidin protein has been identified using immuno-histochemistry and mRNA by real-time PCR but not by *in situ* hybridisation raising the question of whether there is measurable transcription of the hepcidin gene in the central nervous system. Alternatively hepcidin could be transported as a hormone to the brain via the circulation.

**Results:**

By RT-PCR hepcidin mRNA was present at low level throughout normal rat brain while *in situ* hybridisation to detect low-abundant mRNA revealed that transcripts were restricted to endothelium of blood vessels and choroid plexus. In contrast, hepcidin protein analysed by immuno-histochemistry was highly expressed in blood vessels, in endothelium and in pericytes. Hepcidin was also present in glial cells and in the olfactory bulb, sub-ventricular zone and dentate gyrus, areas where neurogenesis and synaptic plasticity are maintained throughout adult life. The hepcidin species identified by Western blotting in sub-ventricular zone, cortex and hippocampus migrated as a ~2.8 kDa band, identical in size to hepcidin present in normal rat serum suggesting that hepcidin in brain was the full-length biologically active 25 amino acid peptide. Hepcidin co-localised with ferroportin in ependymal cells of the sub-ventricular zone and in the corpus callosum consistent with a regulatory role in iron metabolism at these sites.

**Conclusions:**

Hepcidin protein was widely expressed in brain parenchyma while levels of hepcidin gene transcription appeared to be below the limits of detection of the *in situ* hybridisation probes. This disparity suggests that not all hepcidin in the brain is transcribed *in situ* and may originate in part outside the brain. The properties of hepcidin as a cationic peptide hormone are reflected in the finding of hepcidin in the walls of blood vessels and in pericytes and glia, cells that may be involved in transporting the peptide into brain interstitium.

## Background

Iron is an essential nutrient for almost all living organisms but in excess is toxic and regulatory mechanisms have evolved to ensure that iron homeostasis is maintained at both the whole-body and cellular levels [1,2]. The liver-derived peptide hormone hepcidin is the principle regulator of systemic iron homeostasis in mammals [3-5] where it controls the flow of iron into the circulation from multiple sites including duodenal enterocytes, iron-recycling macrophages and hepatocytes [6]. Hepcidin is synthesised by hepatocytes in response to iron signals, inflammation, hypoxia and endoplasmic reticulum stress [7]. Following proteolytic processing [8], the active hormone is distributed in the circulation to cells expressing the hepcidin receptor, ferroportin, which is the only known iron exporter expressed by mammalian cells [9]. Hepcidin triggers the internalisation and lysosomal degradation of ferroportin to regulate iron export [10].

The blood brain barrier in adult animals does not allow the passage of free iron and the majority of iron entering the brain does so through the binding of diferric transferrin to transferrin receptors (TfR1) expressed on the luminal surface of brain capillary endothelial cells (BCECs) [11]. Following receptor-mediated endocytosis iron is released from transferrin and transported across the endosomal membrane or released from the BCECs into brain parenchyma [12]. Recently a model of iron transport across the BBB has been suggested [13] in which the capillary endothelium induces underlying astrocytes to produce ferroxidase activity to support ferroportin-mediated iron efflux by the BCECs. Iron is essential for growth and development of the embryonic brain [14] and in the adult for synthesis of key enzymes, myelin and neurotransmitters. Recently a key role for iron in neuronal function has been identified with the finding that iron is essential for synaptic plasticity and the associated generation of post-synaptic Ca^2+^ signals [15].

How brain iron uptake is regulated and whether hepcidin has a role is uncertain. The constitutive loss of hepcidin through gene mutations in either human [16] or mouse hemochromatosis [17,18] does not appear to cause abnormalities in brain iron levels; this raising the question of whether there is redundancy in the function of hepcidin in the central nervous system. We should also consider whether the functional properties of hepcidin as a hormone, those of production at a remote site (the liver), with transport in the circulation to target tissues are relevant to a potential role for the peptide in brain iron homeostasis. If this is the case there would be the requirement for hepcidin to cross the blood brain barrier to gain access to brain parenchyma. It is known that conditional inactivation of the hepcidin gene in mouse liver recapitulates the severe iron overload seen in the model with total hepcidin knockout [19], this demonstrating that the hepatocyte is the major site of hepcidin synthesis and that other tissues are unable to compensate. Indeed, there is uncertainty over whether hepcidin is synthesised in the brain as mRNA was not detected in early studies by Northern analysis [4,5] and in-situ hybridisation similarly failed to produce a consistent signal [20]. Hepcidin mRNA was detected in normal brain [20,21] and in the presence of systemic inflammation [22] using the highly sensitive technique of RT-PCR but again transcripts were not consistently found in all studies [23,24]. In contrast, hepcidin as an immunoreactive, 25 amino acid (aa) peptide has been reported to be expressed by neurones and astrocytes in the normal mouse brain [20] and also induced by experimental inflammation in the brain [22]. We have recently confirmed that hepcidin protein is widely expressed in all glial cells in normal mouse brain and co-localises with ferroportin in white matter tract [25]. Taken together the results of these studies suggest that there are discrepancies between the low levels of hepcidin mRNA transcripts in regions of normal brain, and the robust expression of the biologically active 25 aa peptide.

In the present study we report a detailed analysis of hepcidin expression in normal rat brain to investigate levels of mRNA and the active 25 aa peptide in neuronal and glial cells. To detect low-abundant mRNA a radioactively-labelled oligonucleotide probe was designed and the results compared with those obtained using a DIG-labelled probe and RT-PCR. The distribution of the mature hepcidin peptide in different brain regions was analysed using highly specific antibodies and appropriate cellular markers. Our results show that hepcidin mRNA was found only in the vascular endothelium including choroid plexus, while hepcidin protein was present throughout all brain cells. The finding of abundant hepcidin protein in the brain parenchyma in the presence of low levels of gene transcription is consistent with an origin for hepcidin outside the brain.

## Results

### Hepcidin mRNA expression in rat brain

**Figure 1:**
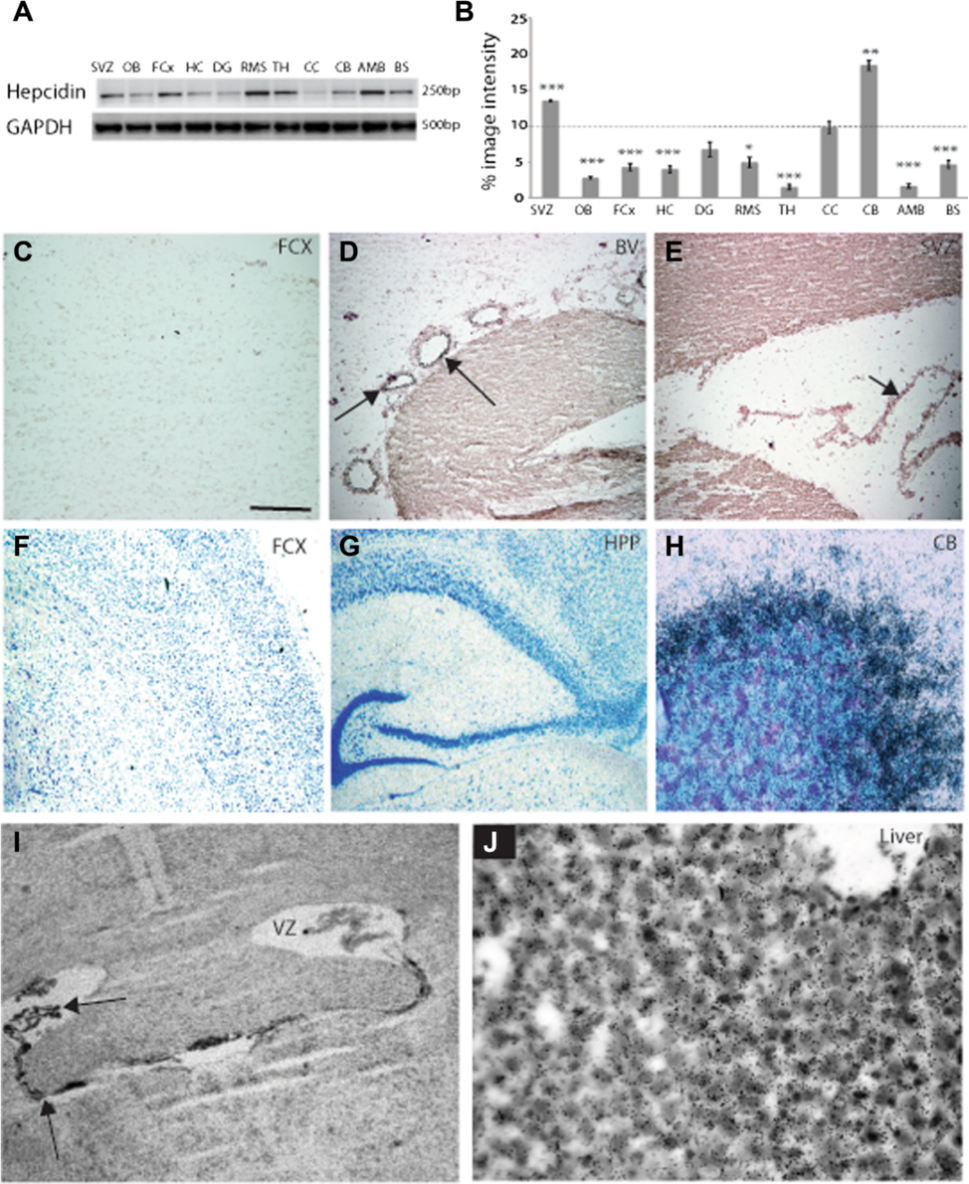
Hepcidin mRNA expression analysed by RT-PCR and *in situ* hybridisation. **A**: Analysis of hepcidin mRNA expression by RT-PCR in adult rat brain (n = 3). Gel banding pattern was - sub ventricular zone (SVZ), olfactory bulb (OB), frontal cortex (FCx), hippocampus (HC), dentate gyrus (DG), corpus callosum (CC), cerebellum (CB) amygdala (AMD), thalamus (TH), choroid plexus (CP) and brain stem (BS). GAPDH was used as loading control. **B**: Graph shows the percentage of image grayscale intensity above background (mean of three samples). Statistical significance compared to whole brain control (dashed line). * = p < 0.05, ** = p < 0.01, *** = p < 0.005. Hepcidin mRNA expression was analysed by *in situ* hybridisation using an antisense DIG-labelled hepcidin probe. mRNA was not visible in cortex **(C)**, and only seen in blood vessels, and choroid plexus **(D** and **E**, localisation of hepcidin mRNA indicated by arrows). To detect low-abundant mRNA an antisense radioactively labelled ([^35^S]-dATP) probe was applied to coronal sections of rat cortex, hippocampus and dentate gyrus **(F** and **G)**. Hepcidin mRNA was not detectable in cortical neurons. Probe activity was confirmed by the finding of a strong signal using an antisense β-actin probe applied to a section of rat cerebellum **(H)**. Hepcidin mRNA was restricted to choroid plexus when the radioactive probe was applied to a section of human brain (**I,** indicated by arrows). A strong signal was detected on a section of rat liver included as a positive control **(J)**. The scale bar in **C** represents 50 μm in **G**; **C** to **F** = 100 μm; 25 μm in **H** and **J**, 70 μm in **I**.

Hepcidin mRNA expression was analysed by RT-PCR in specific brain regions (sub-ventricular zone, olfactory bulb, frontal cortex, hippocampus, dentate gyrus, corpus callosum, cerebellum, amygdala, thalamus, choroid plexus and brain stem). This analysis revealed low levels of mRNA throughout the brain with a slightly higher signal observed in the corpus callosum, cerebellum, choroid plexus and sub-ventricular zone (Figure 1A-B).

To investigate the cellular localisation of hepcidin mRNA *in situ* hybridisation experiments were performed using a full-length probe (350 bp) amplified by DIG labelling. In adult rat brain no signal was detected in the cortex (Figure 1C). A low-intensity signal was consistently observed in blood vessels (Figure 1D) while a clear signal was seen in choroid plexus (Figure 1E).

In order to detect low-abundant mRNA a radioactively-labelled oligonucleotide probe was designed, this showing strong hepcidin expression in adult rat liver (Figure 1J). Even with the use of this probe, however, hepcidin mRNA was not detected in cerebral cortex, hippocampus or dentate gyrus (Figure 1F-G). A β-actin probe used as a positive control showed strong expression in rat cerebellum (Figure 1H). A sense hepcidin probe used to detect non-specific binding produced no visible signal (data not shown). In agreement with these findings mRNA was not detected in cortex or cerebellum from normal human subjects (data not shown), while a clear signal was again present in choroid plexus (Figure 1I). Taken together these *in situ* data suggest that hepcidin transcription in neurons and glial cells in normal brain is below the detection limit of the method. The endothelium of blood vessels and choroid plexus (Figure 1E&I) demonstrated a low level of expression, this likely explaining the signal seen by RT-PCR in corpus callosum and sub-ventricular zone, regions of the brain with a rich vascular supply.

### Molecular characterisation of hepcidin protein in the brain

**Figure 2:**
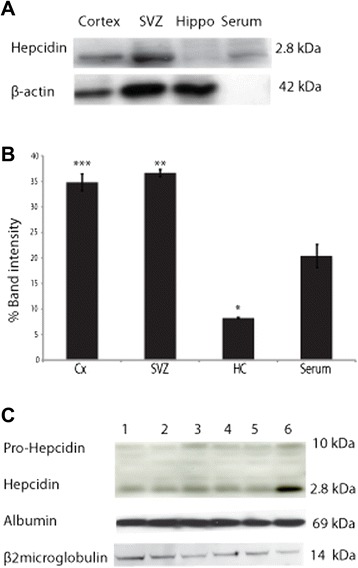
Hepcidin protein expression in brain and serum samples. **A**: Western blotting using a rabbit anti-hepcidin antibody revealed a ~ 2.8 kDa band consistent with hepcidin protein. Lane 1: cortex 2: sub-ventricular zone, 3: hippocampus and 4: rat serum. The highest intensity was seen in the sub-ventricular zone and cortex and lowest in hippocampus. Hepcidin was detected in serum as a band of same molecular mass as seen in brain tissues. A β-actin loading control was used to normalize data in brain tissue, this protein was absent from serum as expected. **B**: Graph shows the percentage of band intensity normalized to loading control (n = 3), * = p < 0.05, ** = p < 0.01, *** = p < 0.005. **C:** In human serum (lane 1–5) hepcidin was seen as a clear ~ 2.8 kDa band while in CSF (lane 6) hepcidin was very strongly expressed. A weak ~ 10 kDa pro-hepcidin band was seen in all human samples. An anti-albumin antibody and anti-β2Microglobulin antibodies were used as loading controls. These are single experiments to assess the antibody specificity and no statistical evaluation was performed.

Hepcidin protein was quantified in homogenates prepared from different brain regions and normal rat serum by Western blotting using a specific rabbit anti-hepcidin antibody. In the case of both brain lysates and serum samples 20 ug of protein was loaded in each lane. This analysis revealed a ~ 2.8 kDa band with highest intensity in the sub-ventricular zone and cortex and lowest in the hippocampus (Figure 2A-B). Hepcidin was detected in rat serum as a band of the same molecular mass as seen in brain tissue (Figure 2A). As a loading control for the brain samples a β-actin antibody was used to re-probe the blot and this allowed a relative comparison of hepcidin levels in different regions of the brain (Figure 2B). As β-actin is not present in serum (Figure 2A) a direct comparison of hepcidin in brain and serum was not possible but as the same amount of protein was added (20 ug) we conclude that per unit weight of protein hepcidin is at least as abundant in the cortex and sub-ventricular zone as in serum. In human serum and CSF hepcidin was also detected as a 2.8 kDa band together with a minor 10 kDa band representing pro-hepcidin (Figure 2C). Hepcidin was highly expressed in CSF. On this gel two antibodies recognising an abundant serum protein (albumin) and a less abundant protein (β2microglobulin) were used as loading controls.

### Cellular localisation of hepcidin protein in the brain

**Figure 3:**
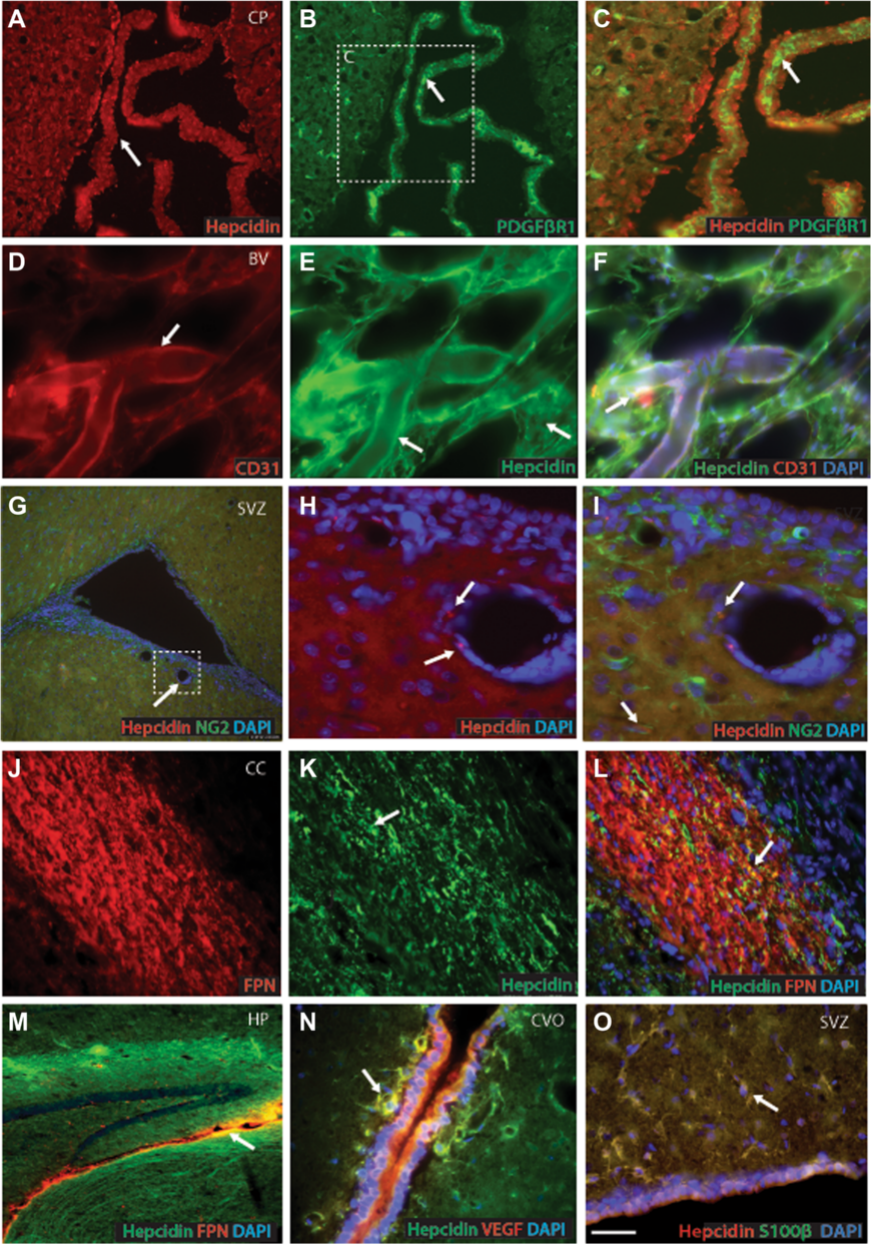
Localisation of hepcidin protein in rat brain endothelial cells. Double immunofluorescence (IFC) staining was performed with polyclonal rabbit anti-hepcidin and other monoclonal antibodies with counterstaining for nuclei with DAPI (Blue). Hepcidin protein was expressed in epithelial cells of the choroid plexus where minimal co-localisation with the pericyte marker PDGFβR1 was observed (arrow in panel **A-C)**. At higher magnification hepcidin was visible in the endothelial lining of blood vessels where it co-localised with the endothelial marker CD31 **(D-F)**, but not with the glial marker chondroitin sulphate proteoglycan neuron ⁄ glia antigen 2 (NG2) **(G-I)**. At higher magnification hepcidin protein was visible in blood vessel walls **(H-I)**. Hepcidin protein was seen in the corpus callosum with some co-localisation with ferroportin noted **(J-L)**, and a similar finding was seen in a section of third ventricle **(M)**. Hepcidin was present in the vascular endothelium of the circumventricular organs where co-localisation with VEGF was seen **(N)**. In the sub-ventricular zone hepcidin was co-localised with S100β **(O)**. Protein expression and co-localisation was indicated with arrow in relevant panels. The scale bar in **O** represents 70 μm in **A** and **B** and M; 20 μm in **D-F**, **H** and **I**; 50 μm in **G**; 30 μm in **C, J-N** and **O**.

In brain sections analysed by immunohistochemistry and viewed under low power, hepcidin immuno-reactivity was associated with the choroid plexus and blood vessels. Hepcidin protein was present in the epithelial cells of the choroid plexus but there was limited co-localisation with the pericyte marker PDGFβR1 (Figure 3A-C). At higher magnification hepcidin was visible in the endothelial lining of blood vessels where it co-localised with the endothelial marker CD31 (Figure 3D-F). Hepcidin protein was present in the sub-ventricular zone close to blood vessels (Figure 3G) and did not co-localise with the glial marker chondroitin sulphate proteoglycan neuron⁄glia antigen 2 (NG2) (Figure 3G-I). At higher magnification (panels H-I) hepcidin protein was present in endothelial cells of blood vessels. There was clear co-localisation between hepcidin and ferroportin in the corpus callosum (Figure 3J-L) and also in the ependymal cells of the sub-ventricular zone (Figure 3M). Additional evidence for the presence of hepcidin in vascular endothelium was the finding of co-localisation with the endothelial marker vascular endothelial growth factor (VEGF) in circumventricular organs (CVO) (Figure 3N). Although hepcidin did not co-localise with the mature glial marker (NG2) this was demonstrated with S100β indicating that the peptide is expressed in glial precursor cells (Figure 3O).

**Figure 4:**
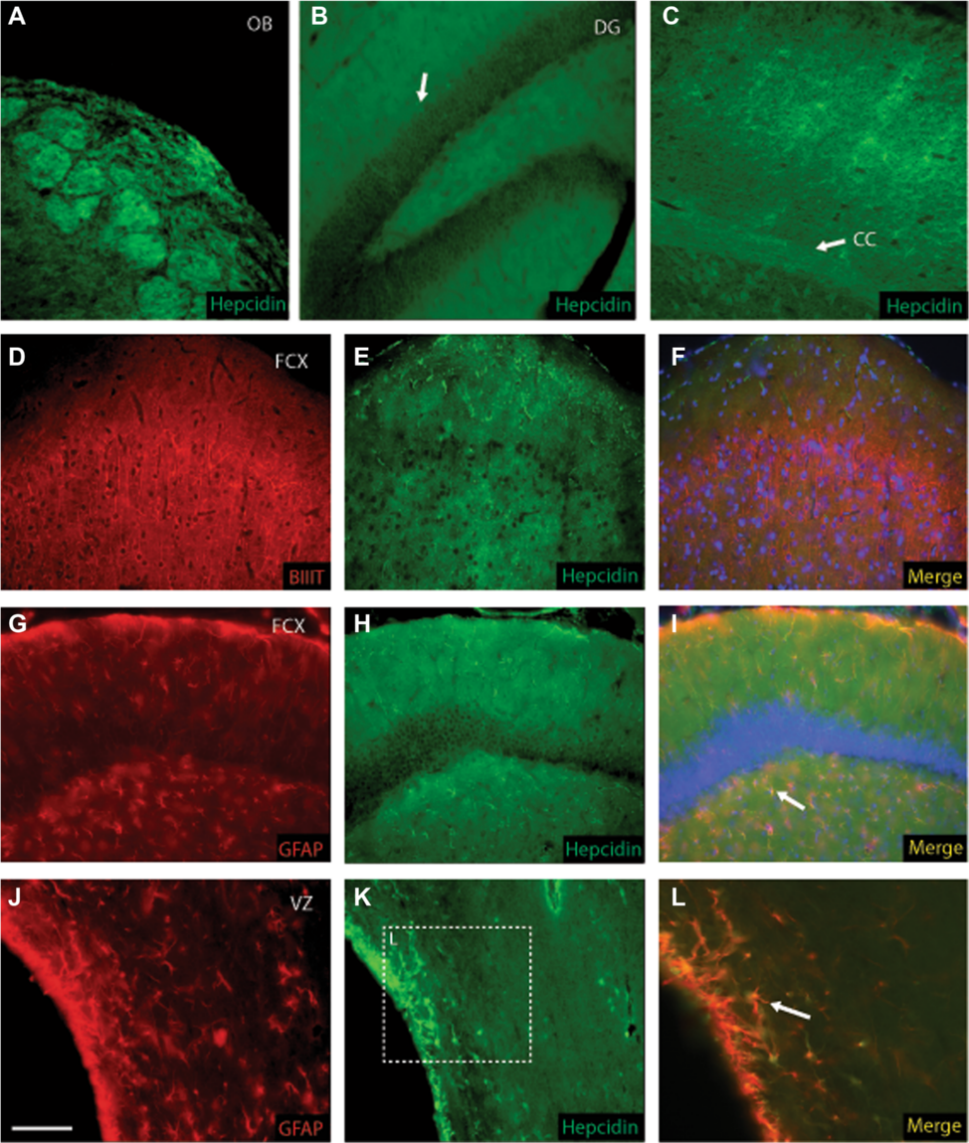
Cellular localisation of hepcidin protein in rat brain in neurons, astrocytes and endothelial cells. Hepcidin protein was visible in the molecular layer and glomerular cells of the olfactory bulb **(A)**, dentate gyrus granule cells **(B)**, white matter tracts of corpus callosum, cortex and striatum **(C)**. Hepcidin was present in the upper layers of the cortex but limited expression was observed in β3 tubulin-positive neurons **(D-F)**. Hepcidin expression was seen in the cortex mainly in astrocytes and CA region of the hippocampus with co-localisation noted in GFAP positive cells **(G-I)**. Hepcidin was present in the ependymal cells of lateral ventricle (arrow) and cells within the subventricular zone where co-localisation with GFAP in the wall of the ventricle was observed **(J-L)**. Protein expression and co-localisation was indicated with arrow in relevant panels. The scale bar in **J** represents 50 μm in **A** to **C, J and K**; 100 μm in **D-I**; 25 μm in **B and L**.

Hepcidin was strongly expressed in the olfactory bulb, granule cells of dentate gyrus and as noted above in the white matter tract of corpus callosum, cortex and striatum (Figure 4A-C). Hepcidin protein was also seen in the cortex and hippocampus but co-localisation with the mature neuronal marker βIII-tubulin was not observed (Figure 4D-F). In contrast co-localisation with the mature astrocytic marker GFAP was seen confirming the presence of hepcidin in glial cells (Figure 4G-I). Hepcidin protein was visible in the CA region of the hippocampus (Figure 4H). Hepcidin protein was strongly expressed in the walls of the lateral ventricles where it co-localised with GFAP in astrocytic end feet in close proximity to the ependyma (Figure 4J-L, marked with arrow).

## Discussion

The aim of this study was to investigate the expression of hepcidin mRNA and protein in normal rat brain and to interpret the findings in the light of the properties of hepcidin as a peptide hormone with an essential role in systemic iron homeostasis. A member of the defensin family of cationic antimicrobial peptides [26], hepcidin is synthesised in the liver and transported in the circulation to target tissues where it controls the iron export function of the ferrous iron permease, ferroportin [10]. The finding that hepcidin protein is present in brain interstitium, specifically in neurons and glial cells [20] raises the question of whether the peptide is produced in situ, by transcription of the hepcidin gene, HAMP, or transported to the brain in the circulation in the manner of a classical hormone. Hepcidin mRNA was reported to be present in normal mouse brain using real-time PCR [20,21] and to be induced in rat brain by the systemic administration of lipopolysaccharide where the increase in hepcidin transcripts was limited to the cortex and substantia nigra [22]. A transitory increase was also observed in mouse and rat choroid plexus 3 hours after the administration of lipopolysaccharide [27]. While hepcidin transcripts were consistently found during inflammation in this report, they were rarely detected under basal or control conditions. Other studies have reported an increase in hepcidin mRNA levels in the cortex, hippocampus and striatum in a rat model of cerebral ischemia [28] and in mouse brain following systemic bacterial challenge [29]. In the present study we found that hepcidin mRNA could be detected by real time PCR at a low level throughout normal brain with a slightly higher signal found in the corpus callosum, choroid plexus and frontal cortex.

Real-time PCR is a highly sensitive technique that provides information on transcript levels in relatively large areas of the brain obtained by anatomical dissection. To investigate hepcidin transcription in more detail the cellular location of the mRNA was analysed by *in situ* hybridisation. Using a digoxigenin-labelled probe a signal was not detectable in cortical areas but a low-intensity signal was visible in blood vessels and a clear signal was seen in the choroid plexus. It has previously been reported using this type of probe that a low-level signal was detectable in specific regions of normal mouse brain including piriform cortex, facial nucleus and granule cells of the cerebellum while a stronger signal was detected in dorsal root ganglia [20]. However, in agreement with the present study a specific mRNA signal could not be detected in most cortical areas suggesting that transcripts were of low abundance and likely to be below the limits of detection of the probe. With the aim of detecting low abundant mRNA a radioactively-labelled probe was synthesised but even with this probe no signal was detectable in cortical areas and transcripts were again limited to blood vessels and choroid plexus. If hepcidin transcription is mostly limited to vascular structures then this could explain the findings obtained by real-time PCR in which low levels of mRNA were detected throughout the brain while a higher signal was observed in regions with a rich vascular supply. Further evidence in support of there being limited transcription of hepcidin mRNA in neurons comes from a study of primary cultures of brain cells using real-time PCR where mRNA was undetectable in neurons under basal conditions and was not induced by the addition of either inflammatory mediators or iron. This was in contrast to cultures of astrocytes and microglia that responded to both conditions with an increase in levels of hepcidin mRNA [30].

In contrast to the limited transcription of the hepcidin gene, hepcidin protein was widely expressed throughout the brain as demonstrated by Western blotting and immunohistochemistry. The hepcidin species identified by Western blotting migrated as a ~2.8 kDa band, identical in size to hepcidin present in rat and human serum suggesting that the species in the brain was the full-length biologically active 25 amino acid peptide. Highest levels were found in the cortex and sub-ventricular zone with lower levels observed in the hippocampus. The anti-hepcidin antibody used in these experiments specifically recognised the 2.8 kDa hepcidin species as opposed to other antibodies that recognise both the 2.8 kDa species and a 10 kDa peptide representing pro-hepcidin. This form of hepcidin lacks biological activity unless it is cleaved to yield the mature 25 amino acid peptide by a furin-dependent process [8] and the presence of pro-hepcidin in brain [31] is of uncertain functional significance.

Using the specific antibody in immunohistochemistry experiments hepcidin protein was strongly expressed in vascular endothelium, choroid plexus and in cortical astrocytes with end-feet that were in close proximity to blood vessels. Hepcidin co-localised with the endothelial markers, CD31 and VEGF, at the luminal aspect of blood vessels but not with the basement membrane marker, NG2. Hepcidin was also detected on the ab-luminal surface of endothelial tubes in pericytes, cells that are important for the development and maintenance of the blood–brain barrier (BBB), regulation of angiogenesis and capillary blood flow, and regulation of the neural response to injury [32]. The presence of hepcidin protein in pericytes suggests a possible role for the peptide in paracrine signaling. The finding of hepcidin in all layers of the walls of blood vessels is consistent with transport of hepcidin across the endothelial cells that make up the BBB. Furthermore, the observation that hepcidin was present in astrocytes intimately associated with the outside of blood vessels suggests that glial cells could be part of a transport pathway providing access to brain interstitium. Hepcidin is a cationic peptide and and evidence showing that cationic proteins readily cross the BBB [33-36] provides further support for the notion that hepcidin can cross the BBB under physiological conditions. The finding that hepcidin was expressed in the circumventricular organs would also be consistent with a role of this organ in transporting hepcidin in to the brain without disrupting the blood–brain barrier. The endothelium of blood vessels and choroid plexus demonstrated low levels of hepcidin mRNA raising the question of whether transcription of the HAMP gene in the brain and systemic vasculature contributes to levels of the circulating peptide. This seems unlikely as it has been reported that conditional inactivation of the hepcidin gene in mouse liver recapitulates the severe iron overload seen in animals with total hepcidin knockout [19]. This suggests that hepatocytes are the major site of hepcidin synthesis and other tissues such as the vascular endothelium produces insignificant amounts of the protein and are unable to compensate for the loss of hepatic production.

Hepcidin immunoreactivity was seen in non-vascular structures with a consistent signal seen in glial cells but not in mature neurons. In agreement with the results of our previous study of normal mouse brain [25] strong hepcidin immuno-reactivity was seen in the granule cells of the dentate gyrus and the sub ventricular zone of lateral ventricle. It is of interest that these two regions are notable for having high rates of neurogenesis in adult rats [37,38] suggesting that hepcidin may have a role in regulating iron metabolism in newly-proliferating cells in the mature brain. Hepcidin was also strongly expressed by oligodendrocytes in all white matter tracts including the rostral migratory stream, corpus callosum, cortico-spinal tracts and cerebellar peduncles, a finding consistent with the transport of this hormone along these tracts together with other small peptide hormones and neurotransmitters [39,40].

## Conclusion

The finding of abundant hepcidin protein in brain parenchyma in the presence of levels of gene transcription that appear to be below the limits of detection is consistent with an origin for at least a proportion of hepcidin outside the brain. The properties of hepcidin as a cationic peptide hormone are reflected in the finding of hepcidin protein in the walls of blood vessels and in pericytes and glia, cells that may be involved in transporting the peptide into brain interstitium.

## Methods

### Animals

Adult Sprague–Dawley (SD) rats (12 weeks old, 180-250 g) were purchased from Charles River. All animals were housed under standard conditions (12 hours light–dark cycle, 20°C ambient temperature) with free access to food and water. All experiments were carried out in accordance with the UK Home Office Regulations for the Care and Use of Laboratory Animals and the UK Animals (Scientific Procedures) Act 1986.

### Tissue preparation

Unfixed tissues were carefully dissected from various brain regions from six adult rat brains and snap frozen in dry ice until analysed by either RT-PCR or Western blotting as described previously [41]. For histochemical analyses, six animals were anesthetised with pento-barbitone, flash perfused transcardially with 0.9% saline followed by 0.1 M phosphate buffer (PBS) with 4% paraformaldehyde (PFA) and the brains cryoprotected as described previously [41]. A freezing microtome was used to prepare 20 μm coronal sections through the entire hippocampus, cortex, mid brains (striatum) and up to brain stem. Sections were then stained by immunohistochemistry as described below.

### Human brain tissues

Human brain tissues from controls (N = 6) were provided by the UK Brain Bank. The Cambridge Health Authorities Joint Ethics Committee for use of human brain tissue gave ethical permission for this study. Autopsy tissue was examined from 6 adults (age (years) 74.0 ± 8.1) as part of another study described previously [42].

### RNA isolation and RT-PCR

For RNA analysis rat brain (n = 3) tissues were dissected from various regions (that also contain blood vessels and endothelium) as described above. RNA was extracted from approximately 50 mg of tissue and 1 ml of TRIzol reagent (Invitrogen) following manufacturer’s instructions and then purified using an RNeasy Mini kit (Qiagen). RNA was treated with DNase I, and cDNA synthesised from 2 μg of the treated RNA using a SuperScript III reverse transcriptase kit (Invitrogen) with random hexamer primers. PCR of the newly synthesized cDNA (8 ng) was performed using PCR Supermix (Invitrogen) with the primer pairs for hepcidin (Forward Primer: CACAGCAGAACAGAAGGCATG,and Reverse primer: CTTCTGCTGTAAATGCTGT, product size 250 bp), using a program of 95°C for 3 min, 30 cycles of 95°C for 30 s, 60°C for 30 s and 72°C for 1 minute. PCR products were separated on 2% agarose gels and photographed under UV illumination. Transcript expression levels were normalised to the expression of GAPDH (Forward Primer: CGGAGTCAACGGATTTGGTCGTAT, Reverse primer: AGCCTTCTCCATGGTGGTGAAGAC, product size 500 bp).

### *In situ* hybridisation (ISH)

Brain tissues were prepared for *in situ* hybridisation and probed as described previously [42]. In addition to sections of rat brains (n = 3), normal human brain sections showing the choroid plexus were analysed. Ethical committee approval for use of this material was documented previously [42]. Briefly, oligonucleotide probes corresponding to rat hepcidin was used for radio-active labelling, hepcidin (Nucleotide 231–277 CTGTAAATGCTGTAAGAATTCCTCCTGTGGTCTCTGTTGCATAAC) was labelled with [^35^S]-dATP (Dupont-NEN) using a 3′-terminal deoxynucleotidyl transferase enzyme kit (Boehringer-Mannheim), hybridization was performed in a humid chamber overnight at 62°C (~16 h). Sections were then washed, dehydrated and air-dried before being exposed to Biomax MR film (Kodak) for 14 days. Non-specific hybridization was abolished in the presence of 100-fold excess unlabelled oligonucleotide. Radioactivity in tissue sections was analysed by dipping slides in Ilford K-5 emulsion (Ilford, UK), followed by storage at 4°C in light proof boxes for 56 days; they were then developed in phenisol (Ilford), fixed and counterstained in 0.2% methylene blue.

### Synthesis of DIG labelled probes for ISH

To synthesize digoxigenin (DIG)-labelled RNA probes, the target hepcidin cDNA was amplified by PCR, using primers designed on the basis of the rat hepcidin cDNA sequence. The primers used for DIG labelling were - (Hepcidin-insF: TAATACGACTCACTATAGGATGGCACTCAGCACTCGGACC Hepcidin-insR: ATTTAGGTGACACTATAGACTATGTTTTGCAACAGATACC). The PCR product was amplified using a 5′ primer containing a T7 phage promoter sequence and a 3′ primer containing an SP6 phage promoter sequence, generating a template for transcription of a sense and an antisense probe, respectively. The PCR products (350 bp) were sequenced and homology checked by BLAST search (NCBI database). *In vitro* transcription reactions were performed using dig-UTP RNA labelling mix (Roche, Mannheim, Germany) and SP6 or T7 RNA polymerase (Roche) following the manufacturer’s instructions.

### ISH with DIG labelled probe

Brain or liver sections were fixed in 4% PFA for 10 min, permeabilized for 10 min in PBS with 0.5% Triton X-100 and acetylated by 10 min incubation in a solution made of 250 mL of water with 3.5 mL of triethanolamine and 625 μL of acetic anhydride added dropwise. Pre-hybridization was performed in hybridization buffer made of 50% formamide, 5 × SSC and 2% blocking reagent (Roche) for 3 hours at 62°C. Hybridization with dig-labelled probes (100 ng/mL) was performed in the same buffer overnight at 62°C. Stringency washing was performed in 0.2 × SSC for 1 hour at 62°C. For the detection of dig-labelled hybrids the slides were equilibrated in maleic acid buffer (0.1 m maleic acid and 0.15 m NaCl, pH 7.5), incubated for 1 hour at room temperature with 1% blocking reagent made in maleic acid buffer (blocking buffer), and then for 1 hour with alkaline phosphatase-conjugated anti-DIG antibodies (Roche) diluted 1:5000 in blocking buffer. The slides were washed twice for 30 minutes in maleic acid buffer and incubated overnight in colour development buffer [2.4 mg levamisole (Sigma), 45 μL 4-nitroblue tetrazolium (Sigma) and 35 μL 5-bromo-4-chloro-3-indolyl-phosphate (Sigma) in 10 mL of a buffer made of 0.1 m Trizma base, 0.1 m NaCl and 0.005 m MgCl_2_, pH 9.5]. The reaction was stopped in neutralizing buffer (0.01 M Trizma base and 0.001 M EDTA, pH 8) and sections mounted in PBS–glycerol and a coverslips applied (Figure 1). Non-specific binding was analysed using sense probes.

### Antibodies

A polyclonal rabbit anti-hepcidin 25 (ab30760) recognising a 2.8 kDa protein [25] was used (1:200 dilution for IHC and IF and 1:100 for WB) and all other antibodies used are listed in Table 1. The following secondary antibodies were used: Alexa Fluor 568-labelled donkey anti-mouse, Alexa Fluor 488-labelled donkey anti-rabbit, and Alexa Fluor 568-labelled donkey anti-goat (all from Invitrogen, 1:1000 dilution for IF).

**Table 1:** **List of the primary antibodies used in this study**

**Antibody**	**Species**	**Dilution**	**Supplier/cat.number**
Anti-hepcidin	Rabbit (polyclonal)	1: 200 for IHC	Abcam (Ab30760)
		1:100 for WB	
Anti-ferroportin	Mouse (monoclonal)	1: 1000 for IHC	Abcam (ab93438)
Anti-GFAP	Mouse (monoclonal)	1:1000 for IHC	Sigma (G3893 Clone G-A-5)
Anti-βIII-tubulin	Mouse (monoclonal)	1:1000 for IHC	Millipore (clone 2G10, neuronal | 05–559)
Anti β-actin	Mouse (monoclonal)	1:10000 for WB	*Sigma (clone AC-74, A5316)*
Anti-CD31	Mouse (monoclonal)	1: 1000 for IHC	Abcam (ab9498)
Anti-PDGFβR1	Mouse (monoclonal)	1: 1000 for IHC	Abcam (ab69506)
Anti-NG2	Mouse (monoclonal)	1: 1000 for IHC	Abcam (ab50009)
Anti-S100β	Mouse (monoclonal)	1: 1000 for IHC	Abcam (ab4066)
Anti-VEGF	Mouse (monoclonal)	1: 1000 for IHC	Abcam (ab46154)
Anti-albumin	Mouse (monoclonal)	1:1000 for WB	Abcam (ab92469)
Anti-β2Microglobulin	Mouse (monoclonal)	1:1000 for WB	Abcam (759)

IHC, immunohistochemistry; WB, Western blot.

### SDS-PAGE and western blotting

Protein lysates were prepared from cortex, hippocampus and sub-ventricular zone of rat brains (n =3), human serum and CSF as described previously [42]. 20 μg protein samples were separated on 10–20% Nu-PAGA Bis-tris (Bis (2-hydroxyethyl)-amino-tris (hydroxymethyl)-methane) gradient gels (1.25 M Bis-Tris pH 6.4, 30% acrylamide/bis), run with NuPAGE® MES SDS running buffer (Lifetechnologies) and transferred to 0.2 μm pore size PVDF membranes (Invitrogen). Membranes were incubated with the appropriate primary antibody in blocking buffer for 24 h at 4°C and then washed three times with 0.1 M tris saline buffer containing 1% Tween 20 (TBST) followed by incubation for 1 hour at room temperature with HRP-conjugated secondary antibodies (anti mouse IgG (1:3000, DAKO) or anti-rabbit IgG (1:3000; DAKO) antibodies). Binding was detected with ECL Plus chemiluminescence reagents and Hyperfilm ECL (both from GE Healthcare). Human serum and CSF were available from a previous study and ethical permission was given as described [25].

### Immunofluorescence (IF)

Sections were blocked using blocking buffer (0.1 M PBS, 0.3% Triton X100, 10% normal donkey serum) for 1 h at room temperature, then incubated overnight at 4°C with primary antibody diluted in blocking buffer. Alexa Fluor-conjugated secondary antibodies were used for detection and samples counterstained with 4′6-diamidino-2-phenylindole (DAPI, Sigma). Sections were then mounted on glass slides with coverslips using FluorSave (Calbiochem).

### Microscopy

Bright field images were taken and quantified using Lucia imaging software and a Leica FW 4000 upright microscope equipped with a SPOT digital camera. Fluorescence images were obtained using a Leica DM6000 wide field fluorescence microscope equipped with a Leica FX350 camera and x20 and x40 objectives. Images were taken through several z-sections and de-convolved using Leica software. A Leica TCS SP2 confocal laser-scanning microscope was used with x40 and x63 objectives to acquire high-resolution images.

### Image and statistics analysis

All experiments were performed in triplicate. RT-PCR and Western blot images were quantified using ImageJ software (US National Institutes of Health), normalising all samples to loading controls. Values in the figures are expressed as mean ± SEM. To determine statistical significance, values were analysed by Student’s *t*-test. A probability value of *p* < 0.05 was considered to be statistically significant.

## Abbreviations

BBB: Blood brain barrier
BS: Brain stem
CP: Choroid plexus
CC: Corpus callosum
CB: Cerebellum
CVO: Circumventricular organs
DAPI: 4′6-diamidino-2-phenylindole
DG: Dentate gyrus
GFAP: Glial fibrillary acidic protein
IHC: Immunohistochemistry
IF: Immunofluorescence
NG2: Chondroitin sulphate proteoglycan neuron ⁄ glia antigen 2
OB: Olfactory bulb
PBS: Phosphate saline buffer
PFA: Paraformaldehyde
PVDF: Polyvinylidene difluoride
RMS: Rostral-migratory stream
SVZ: Sub ventricular zone
TBST: Tris saline buffer with tween
WB: Western blotting
